# Detection of spontaneous anti-neoepitope T-cell responses in non-metastatic bladder cancer patients

**DOI:** 10.3389/fimmu.2025.1627914

**Published:** 2025-11-12

**Authors:** Walther Brochier, Sylvain Nguyen, Valérie Cesson, Orian Bricard, Nicolas van Baren, Gérald Hames, Nicolas Dauguet, Hélène Dano, Bertrand Tombal, Sonia-Cristina Rodrigues-Dias, Audrey Masnada, Raphael Genolet, Alexandre Harari, Beat Roth, Ilaria Lucca, Denise Nardelli-Haefliger, Pierre G. Coulie, Laurent Derré

**Affiliations:** 1 de Duve Institute, UCLouvain, Brussels, Belgium; 2 Department of Urology, Cliniques universitaires Saint-Luc, Brussels, Belgium; 3 Urology Research Unit and Urology Biobank, Department of Urology, University Hospital of Lausanne, Lausanne, Switzerland; 4 Ludwig Institute for Cancer Research, University of Oxford, Oxford, United Kingdom; 5 Ludwig Institute for Cancer Research, Department of Oncology, Lausanne University Hospital (CHUV) and University of Lausanne (UNIL), Lausanne, Switzerland; 6 Department of Urology, University Hospital of Bern, Inselspital, University of Bern, Bern, Switzerland

**Keywords:** neoepitope, bladder cancer, BCG treatment, T lymphocyte, immunotherapy

## Abstract

**Background:**

Bladder carcinomas are immunogenic, and patients with bladder cancer benefit from immune checkpoint therapy. This is correlated to a high tumor mutation burden, which provides a higher number of neoepitopes that can be recognized by tumor-specific CD8^+^ T cells. Intravesical Bacillus Calmette-Guérin (BCG) is used to treat non-muscle invasive bladder cancer (NMIBC), but its mechanism of action remains elusive. Most lymphocytes appearing in the urine of BCG-treated patients are CD4^+^ T cells though preclinical studies showed that CD8^+^ T cells are also necessary for BCG treatment efficacy. It is currently unknown which proportion of patients with non-metastatic bladder cancer develop a spontaneous antitumor CD8^+^ response, and if BCG treatment influences this response.

**Methods:**

In a first cohort of 15 NMIBC and 9 muscle invasive bladder cancer patients, we used IFN-*y* ELISPOT assays to screen for the presence of anti-neoepitope CD8^+^ T cells in the blood, tumor and urine. In a second cohort of 4 NMIBC patients, we analyzed the features and specificity of CD8^+^ T cells infiltrating the tumoral or bladder tissues before and after BCG using single cell transcriptomic analyses. A total of 31 tumor-infiltrating CD8^+^ clonotypes were screened against neoepitopes and tumor cDNA libraries.

**Results:**

9 out of 24 patients from the first cohort mounted a spontaneous and functional anti-neoepitope T-cell response in blood and/or tumor. In 5 patients from this cohort who were treated with BCG, no neoantigen-specific T cells were detected in urine during treatment. In the second cohort, 6 out of 6 TCRs from exhausted CD8^+^ TILs from one patient recognized 5 different neoepitopes. T-cell receptor (TCR) repertoire analyses indicated that the frequencies of these tumor-specific T cells did not increase after BCG instillations, neither in the bladder nor in the blood. None of the 25 other TCRs of CD8^+^ T cells recognized tumor-specific antigens.

**Conclusions:**

We show that one third of patients with non-metastatic bladder cancer mount a spontaneous and functional anti-neoepitope CD8^+^ T-cell response detectable in blood or tumor. In 4 patients with NMIBC, BCG treatment did not boost or induce the anti-neoepitope response, suggesting alternative mechanisms of action for its efficacy.

## Introduction

Bladder cancer (BCa) is the 10th most commonly diagnosed cancer worldwide (1). The majority of BCa patients are diagnosed with non-muscle-invasive BCa (NMIBC) and initially undergo transurethral resection of the bladder tumor (TURBT). For high-risk patients (characterized by the presence of carcinoma *in situ* and/or high-grade Ta/T1 disease), the standard adjuvant treatment consists of repeated intravesical instillations of Bacillus Calmette-Guérin (BCG) once a week for 6 weeks, which reduces both the risk of recurrence (2) and progression to muscle-invasive disease (3). BCa patients can also benefit from immune checkpoint therapy (ICT). PD-L1 blockade is currently the standard maintenance therapy for patients with locally advanced or metastatic bladder cancer who have responded to platinum-based chemotherapy (4). ICT has shown great promise at all stages of the disease, including BCG-unresponsive disease (5), in the neoadjuvant setting (6), and more recently as a first-line treatment for metastatic disease in combination with enfortumab vedotin (7). As in other cancer types, response to ICT is often associated with a high bladder tumor mutation burden, which generates neoepitopes that can be recognized by tumor-specific cytolytic CD8^+^ T cells (8). One of the key mechanisms of action of ICT is the amplification of pre-existing tumor-specific CD8^+^ T cells. In BCa, the identification of neoantigen-specific T cells has been poorly investigated, especially in early stages of BCa. Guéguen et al. characterized the first bladder neoantigen-specific CD8^+^ T cell from the blood of one MIBC patient (9). In addition, Leko et al. analyzed TILs from five patients with non-metastatic bladder cancer and screened them for recognition of predicted neoepitopes. In only one patient, they identified a CD4^+^ T-cell clone recognizing a peptide encoded by the mutated gene *CTBP1^Q277R^*, but no neoepitope-specific CD8^+^ T cells were detected (10). More recently, spontaneous anti-neoepitope CD8^+^ T-cell responses have been detected in the blood of 18 out of 24 patients with metastatic BCa (11). However, the prevalence of such responses and their correlation with clinical outcomes in earlier stages of BCa remain unknown.

BCG instillations elicit a complex immune response involving both the innate and adaptative immune systems (12, 13). In mice, the therapeutic efficacy of BCG has been shown to depend on both CD4^+^ and CD8^+^ T cells, as depletion of either population abolishes the treatment effect (12). Biot et al. observed in a murine model that prior exposure to BCG significantly enhanced treatment efficacy, involving BCG-specific CD4^+^ and CD8^+^ T cells (14). Antonelli et al. further showed that the anti-tumor effect of BCG was mainly driven by a tumor-specific immune response (15). However, in patients, the mechanism of action of BCG therapy in bladder cancer remains poorly understood, particularly regarding how tumor cells are eliminated. Given the antigenicity of bladder carcinomas and their recognition by anti-neoepitope CD8^+^ T cells, it is compelling to investigate the role of tumor-specific cytolytic CD8^+^ T cells during BCG treatment.

In this study, we examined the prevalence of anti-neoepitope CD8^+^ T cells in the blood and tumors of 24 patients with non-metastatic BCa, as well as in urine of five of these patients. Additionally, we assessed the features and tumor specificity of CD8^+^ T cells from blood and bladder tissues of four other NMIBC patients before and after BCG instillations.

## Materials and methods

### Patients and sample collection

Two cohorts of patients participated in this observational study. The first cohort consisted in 15 NMIBC and 9 MIBC patients recruited at the Lausanne University Hospital (**Table 1**). All NMIBC patients were treated by TURBT and 9 (BCG-naïve patients) of them received a subsequent BCG treatment. All MIBC patients were treated by cystectomy and 2 of them received neoadjuvant chemotherapy. The second cohort consisted in 4 patients diagnosed with NMIBC, recruited between February and July 2020 at the department of urology of the Cliniques universitaires Saint-Luc in Brussels. They were surgically treated and received BCG treatment. The study protocol was approved by the ethics committee of the hospital (Belgian registration n°B403201938826) and of Canton de Vaud (Switzerland; #2019-00546). All patients provided written informed consent before enrollment in the study.

**Table 1:** Cohort 1 patient characteristics.

Characteristics	All patients	Patients with detectable neoantigen-specific CD8^+^ T cells	Patients without detectable neoantigen-specific CD8^+^ T cells	P value
No. of patients	24	9	15	
Age (yr), median (min-max)	72 (47–91)	74 (47-87)	67 (56-91)	ns (P = 0.55)^d^
Sex, *n*				
Male	19	7	12	ns (P = 0.99)^e^
Female	5	2	3	
Tumor stage				
NMIBC low grade^a^	3	0	3	ns (P = 0.23)^e#^
NMIBC high grade^b^	12	6	6	
MIBC^c^	9	3	6	
Number of mutations/megabase, median (min-max)	5.1 (0.59-16.68)	6.7 (0.82-16.68)	2.8 (0.59-6.89)	P = 0.018^d^

^a^pTaLG; ^b^pTis, pTaHG, pT1; ^c^pT2-4.

^d^Mann Witney test; ^e^Fisher’s exact test; ^#^Comparison between low and high grade.

For the patients of cohort 1, peripheral blood was collected before TURBT or cystectomy and peripheral blood mononuclear cells (PBMCs) were isolated by density gradient centrifugation and cryopreserved, as described (16, 17). Finely minced fresh bladder tumors were cultured in RPMI with 8% of human serum (HS) and recombinant human (rh) IL-2 at 6,000 U/ml for three weeks. From the established tumor infiltrating lymphocyte (TIL) cell lines, a fraction of the cells was further amplified over 14 days with a rapid expansion protocol (REP) (17). For NMIBC patients undergoing BCG treatment, urine samples were obtained after each instillation to expand urinary T cells with REP, as described (16).

For the patients of cohort 2, bladder tumor samples, healthy bladder tissues and PBMCs were collected during TURBT (pre-BCG) and after BCG treatment (i.e. 6 weeks after the sixth BCG instillation; post-BCG). For tissue samples, histological analyses confirmed that tumor cells were present in all tumoral pre-BCG samples and absent from the non-tumoral pre-BCG samples and from the post-BCG samples. Tissue samples were finely minced and mechanically dissociated with a gentleMACS dissociator (Miltenyi), then passed through a 40 µM filter and washed in PBS 1% HS 2 mM EDTA. All samples were then labelled for 30 min at 4°C with an antibody panel (**Supplementary Methods**) prepared in 10 µl Brilliant Stain buffer plus (BD Biosciences, #566385). Samples were acquired on a FACSAria III Cell Sorter (BD Biosciences) and single CD8^+^ T cells were sorted in 384-well plates containing 1 µl per well of capture mix, composed of RNase-free H_2_0, 2.5 mM dNTP (ThermoFisher Scientific, #R0192), 0.5% Triton X100 (Sigma, #T8787), 125U recombinant RNase Inhibitor (Takara, #2313A) and 2.5 µM reverse-transcription oligonucleotide, and frozen at -80°C. Single cell RNA-seq libraries were prepared from thawed sorted single cells using the Smartseq2 protocol, in order to extract TCR sequences. Libraries were sequenced on an Illumina HiSeqX (Macrogen Europe). Reads were aligned to human reference genome hg38 using STAR and sorted and indexed with SAMtools. Gene expression quantification was obtained with htseq-count. *TRA* and *TRB* sequences were extracted using Mixcr3 (18). Data analysis was performed in R/Bioconductor as described elsewhere (19).

### Neoepitope prediction and testing for patients of cohort 1

Whole-exome-sequencing (WES) libraries were prepared using the xGen Dual Index UMI Adapters (IDT), and genomic DNA (gDNA) extracted (DNeasy kit, Qiagen) from formalin-fixed paraffin-embedded (FFPE) or snap-frozen tumor tissue, as well as autologous PBMCs or TILs. Libraries were subjected to paired-end sequencing using Illumina HiSeq4000 with a coverage depth of 250X. The exome reads were aligned to the human reference genome hg38, followed by somatic variant calling using a bioinformatics pipeline consisting of MicMap (https://github.com/sib-swiss/micmap ) and the Variant Effect Predictor (VEP) tool (https://www.ensembl.org/info/docs/tools/vep/index.html). Library preparation was carried out with the AllType FASTplex NGS 11 Loci Flex Kit (One Lambda), and sequencing was conducted on the Illumina MiSeq platform. The binding affinity of 8- to 11-mer candidate mutated peptides to their corresponding HLA class I molecules was assessed using PRIME v1.031814 (20). For each patient, up to 150 peptides with the lowest PRIME %Rank scores were synthesized at the Protein and Peptide Chemistry Facility of the University of Lausanne.

For neoepitope antigenicity evaluation in TILs, IFN-γ-secreting TILs were assessed using an IFN-γ ELISpot assay (Diaclone) upon stimulation with a pool of neoepitope candidates. Neoepitope was considered antigenic if it induced an average number of IFN-γ-secreting cells exceeding the negative control by more than three times the standard deviation and was confirmed in ≥2 independent experiments. For urinary T cells, CD8^+^ cells were magnetically isolated, underwent a second REP protocol before a challenge with a pool of predicted neoepitope peptides followed by IFN-γ ELISpot. For neoepitope antigenicity evaluation in PBMCs, CD8^+^ T cells were magnetically isolated and stimulated for 14 days with autologous irradiated CD4^neg^CD8^neg^ fractions and a pool of predicted neoepitope peptides in RPMI supplemented with 8% HS and rec-hu-IL-2 (20 IU/mL for the first 2 days, followed by 150 IU/mL). The cells were then restimulated overnight with the same pools of peptides and irradiated autologous CD4^+^ blast cells (21), and their reactivity against neoepitope peptides was evaluated by IFN-γ ELISpot assays. The recognition of wild-type (WT) peptides by neoepitope-specific T cells was assessed by IFN-γ ELISpot assays in the presence of neoepitope or WT peptide dilutions.

To test neoepitope-reactive T-cell polyfunctionality, TILs or PBMCs (5x10^5^ cells/well) were cultured in RPMI supplemented with 10% FCS, with autologous CD4^+^ blasts (5x10^5^ cells/well) and neoepitope (1 µM) for 6h. After 1h, Protein Transport Inhibitor Cocktail (eBioscience) was added with anti-CD107a-BV605 (H4A3, Biolegend). Surface antigen staining was performed by incubating stimulated cells with a panel of monoclonal antibodies (mAbs) for 20 minutes at 4°C in staining buffer (PBS, 0.2 % bovine serum albumin, 2 mM EDTA), together with the Aqua live/dead stain kit (Thermo Fisher Scientific) and Fc-receptor blocking reagent (Miltenyi). mAbs were used at optimal dilutions and included: anti-CD4-BUV661 (M-T477), anti-CD8-BUV395 (G42-8), anti-CD45-BUV737 (HI30) (BD Biosciences), and anti-CD3-PE/Cy7 (UCHT1) (BioLegend). For intracellular cytokine staining, cells were fixed for 30 minutes at room temperature using the Intracellular Fixation and Permeabilization Buffer Foxp3 set (eBioscience), followed by staining with anti-IFN-γ-BV421 (4S.B3), anti-TNF-α-AF647 (Mab11), and anti-IL-2-PE (MQ1-17H12) (BioLegend). Sample acquisition was performed using the CytoFLEX-LX2 flow cytometer (Beckman Coulter), and data analysis was conducted with FlowJo software.

### Tumor antigen identifications for patients of cohort 2

DNA was extracted from PBMCs or frozen tissue samples. For patient UC4, DNA was extracted from FFPE tissue. For WES, DNA fragment libraries were prepared from gDNA extracted either from frozen tumor fragments or PBMC and were amplified for exon DNA using the SureSelect V7 method (Agilent) for patients UC1, 2 and 3. For patient UC4, the library was prepared from gDNA extracted from frozen or FFPE tumor tissue and PBMC using the Human Core Exome kit (Twist Bioscience). The libraries were paired-ended sequenced on a Novaseq (Illumina), generating 151 bp-long sequences with a coverage depth of 50X (for PBMC) and 200X (for tumor samples) in paired-end mode. The sequenced reads were trimmed and filtered as above. The resulting reads were aligned to the GRCh38 reference human genome sequence using the align function from the Rsubread Bioconductor package (22) in DNA mode and built-in genome annotations.

For bulk RNAseq, poly-A RNA was purified from total RNA extracted from frozen tumor fragments (patients UC1, 2 and 3) and used as template to prepare a cDNA library according to the TruSeq Stranded mRNA method (Illumina). The cDNA libraries were paired-end sequenced on the Novaseq platform (Illumina). The sequenced RNA-seq reads were trimmed with the ShortRead Bioconductor package (23) to remove adapter sequences as well as leading and trailing bases with Phred quality scores <20 and were further filtered to remove reads shorter than 50 bases and unpaired reads. The resulting reads were aligned to the GRCh38 reference human genome sequence using the splice-aware subjunc function from the Rsubread Bioconductor package and the Homo_sapiens.GRCh38.105.gtf genome annotations (Ensembl). Raw read counts per gene were calculated with featureCounts (Rsubread package, with multi-mapping reads reported as fractionated counts) and normalized in TPM.

Somatic variants were identified using Mutect2 (24), Strelka2 (25) and Varscan2 (26) and annotated with Funcotator (GATK4). Only missense variants identified by at least 2 of the 3 variant caller tools were selected. For each variant amino acid and its wild-type counterpart, a 25 amino acid-long sequence spanning the centered position was generated. For each of these sequences, 8 to 11-amino acid-long peptides able to bind to the HLA-A, HLA-B and HLA-C types of the patient were predicted with NetMHCpan4.0 (27). Only peptides derived from a gene expressed at a level >1 TPM as determined by RNA-seq on the same tumor sample and associated with a strong predicted binding affinity (%Rank <0.5%) were selected for synthesis.

The other methods for the analyses of the patients of cohort 2 are further described in **Supplementary Methods**.

## Results

### Presence of neoepitope-specific CD8^+^ T cells in TILs and PBMCs from bladder cancer patients

**Figure 1:**
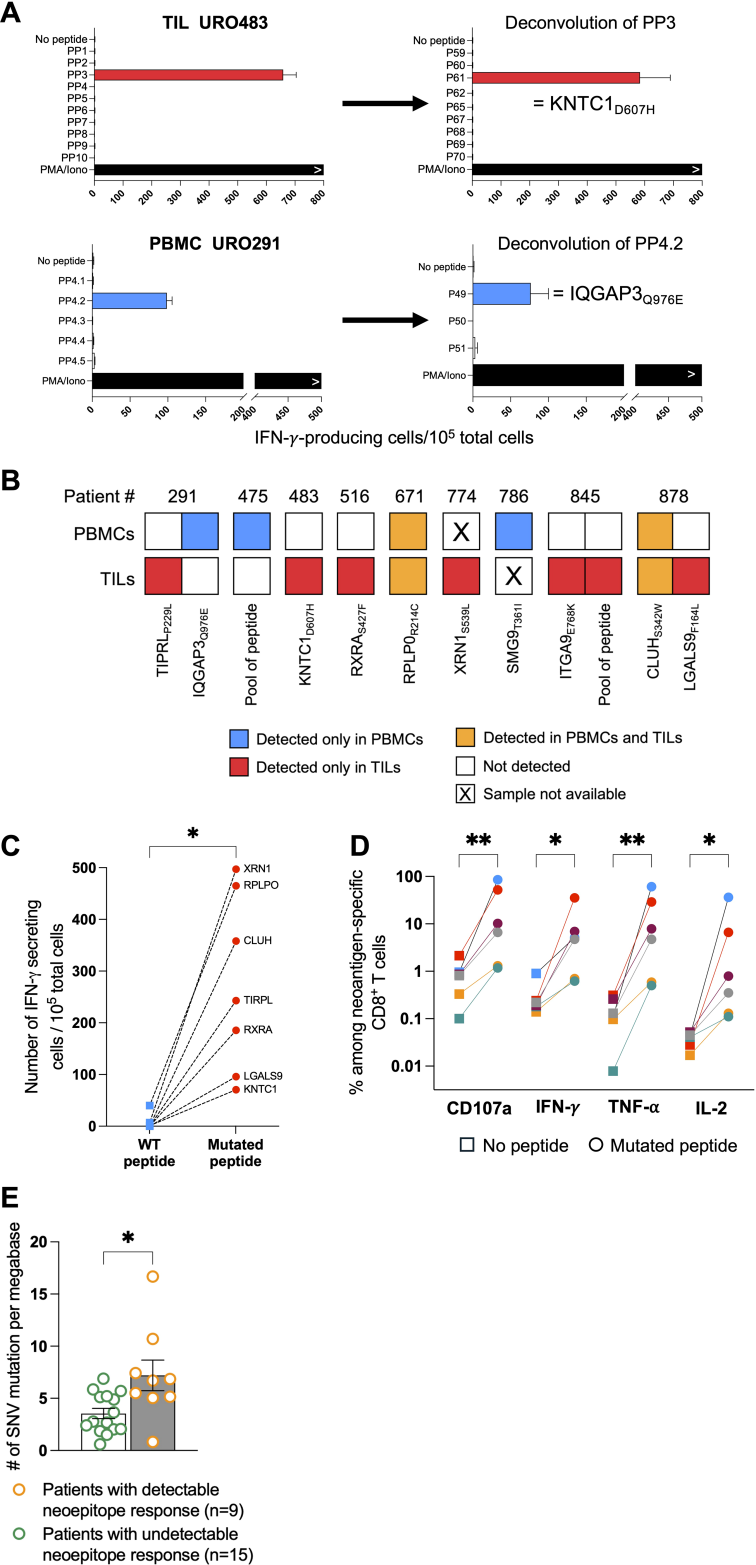
Identification of neoepitope-specific T cells in TILs and PBMCs from bladder cancer patients. **(A)** Representative examples of deconvolution of peptide pools in TILs or PBMCs from two patients (URO483 and URO291). **(B)** Neoepitope repertoire from the 9 patients with neoantigen reactivities. Each colored square represents one neoepitope for which CD8^+^ T-cell reactivity was observed either exclusively in PBMCs (blue), exclusively in TILs (red) or in both compartments (orange). **(C)** CD8^+^ T-cell reactivity from TILs or PBMCs measured by IFN-*y* ELISpot against neoepitope (red dot) and wild-type (WT) non-mutated peptide (blue dot). **(D)** CD107a, IFN-*y*, TNF-α and IL-2 production measured by intracellular labelling upon neoepitope stimulation by TILs from 6 patients (one color per patient). **(E)** Total single-nucleotide variant (SNV)-mutational burden in patients with or without detectable neoepitope-specific CD8^+^ T-cell responses in TILs or PBMCs. Indicated P values were determined by Wilcoxon t-test **(C, E)** and one-way ANOVA **(D)**. Significant differences between paired groups are indicated by *p < 0.05 and **p < 0.01. PP, peptide pool.

We first analyzed a cohort of 24 patients with non-metastatic bladder cancer (**Table 1**). The tumor mutational burden was established from surgical samples. The median of non-synonymous single nucleotide substitutions per tumor was 172.5 with a range of 20-567, which is in line with previous reports (10, 28). No difference was observed when stratifying our cohort according to tumor stage or grade (data not shown). Mutant peptides of 8 to 11 amino acids predicted to bind to autologous HLA class I molecules were obtained with PRIME v1.0318 (20) and the 105–150 best-ranked peptides were synthesized. We used IFN-*y* ELISPOT assays to screen bulk TILs and PBMCs against pools of 10 to 20 mutant peptides (3400 peptides in total). Out of 400 such recognition assays, 14 were positive (**Figure 1A**). It led to the identification of 10 different neoepitopes (**Figure 1B**, **Supplementary Table 1**). Only 3 patients had T cells against neoepitopes in both TILs and PBMCs. In two patients, URO671 and URO878, T cells from both TILs and PBMCs recognized the same neoepitope. Three patients, URO291, URO845 and URO878, had T cells against two neoepitopes. Overall, 9 out of the 24 patients had detectable neoepitope-specific T cells: 6 NMIBC and 3 MIBC patients (**Figure 1B**). Of note, in two patients (URO475 and URO845), we observed recognition of a pool of peptides, but failed to deconvolute to the single peptide, owing to the scarcity of biological materials. Among the identified mutated genes (**Supplementary Table 1**), only *IQGAP3* (29) and *RXRA* (30) mutations have been already observed in cancers. Notably, mutations of the *RXRA* gene can be found in 9% of MIBC and 58% of them consist in S427F/Y mutation (30). For most of all the identified peptides, we verified that the corresponding WT peptide was not or weakly recognized (**Figure 1C**). In addition, upon stimulation by their cognate neoepitope, TILs from 6 patients were shown by flow cytometry to upregulate expression of CD107a, IFN-γ, TNF-α and IL-2, indicating polyfunctionality (**Figure 1D** and **Supplementary Figure 1**). However, we were unable to generate autologous tumor cell lines, so we could not confirm that the anti-neoepitope CD8^+^ T cells recognize autologous tumor.

As expected, the median tumor mutational burden was higher for patients with detectable anti-neoepitope T cells as compared to those without detectable anti-neoepitope T cells (**Figure 1E** and **Table 1**). Furthermore, in contrast to NMIBC patients, MIBC patients harboring detectable spontaneous neoepitope CD8^+^ T-cell responses exhibited improved recurrence-free survival compared to patients without neoantigen responses (**Table 2**, **Supplementary Figure 2**). Of note, no difference in recurrence-free survival was observed when considering only NMIBC patients (n=9; 4 with and 5 without detectable neoantigen-specific T cells) who underwent a BCG therapy (data not shown). Larger cohorts are needed to confirm this result.

**Table 2:** Association between the absence of recurrence/progression and the presence of neoantigen-specific CD8^+^ T cells in MIBC patients.

Characteristics	All patients	Patients with neoantigen-specific CD8^+^ T cells	Patients without neoantigen-specific CD8^+^ T cells	P value
NMIBC patients				
No recurrence	11	4	7	>0.999^b^
With recurrence	4	2	2	
MIBC patients^a^				
No progression	3	3	0	0.0179^b^
With progression	5	0	5	

^a^No clinical follow-up for one patient; ^b^Fisher’s exact test.

Finally, we established 10 T-cell lines from urines collected during BCG treatment from 5 out of 9 patients with NMIBC. They were screened with IFN-γ ELISPOT for recognition of the autologous mutant peptides, with negative results. However, no neoepitope-specific T cells were detected in the TILs or PBMCs of these 5 patients (data not shown).

We conclude from this cohort of 24 patients with non-metastatic bladder cancer screened for the presence of anti-neoepitope T cells, the largest series thus far analyzed in this cancer type, that about one third of patients mounted a spontaneous anti-neoepitope T-cell response detectable in blood or tumor, which might be associated with tumor control in MIBC patients.

### Gene expression profiling of bladder CD8 T cells before and after BCG

Since we did not detect urinary neoantigen specific-CD8^+^ T cells from the BCG-treated patients from cohort 1, we focused on 4 new NMIBC patients with high grade tumors (**Table 3**) to gain more insights on bladder neoantigen-specific T-cell features upon BCG therapy. Thus single CD8^+^ T cells from tumor collected during the TURBT (pre-BCG) and non-tumor tissues collected at the TURBT and after the BCG therapy (post-BCG) were sorted for RNAseq using Smart-Seq2 (31) (**Table 3**).

**Table 3:** Cohort 2 patient characteristics and numbers of single cells processed for RNAseq.

Patients	Sex	Age	Tumor grade and stage	Follow up (months)	Recurrence	Number of mutations per megabase	Number of sorted single CD8^+^ T cells		
							Week 0 (preBCG)		Week 17 (postBCG)
							Tumor	Normal tissue	
**UC1**	M	84	HG pT1	† 18 sept 21	no	11.5	266	158	48
**UC2**	F	73	HG pT1	36	no	2.8	372	0*	172
**UC3**	M	82	HG pTa	32	no	7.4	302	198	206
**UC4**	M	67	pTis	31	no	2.1	57	90	171

**Figure 2:**
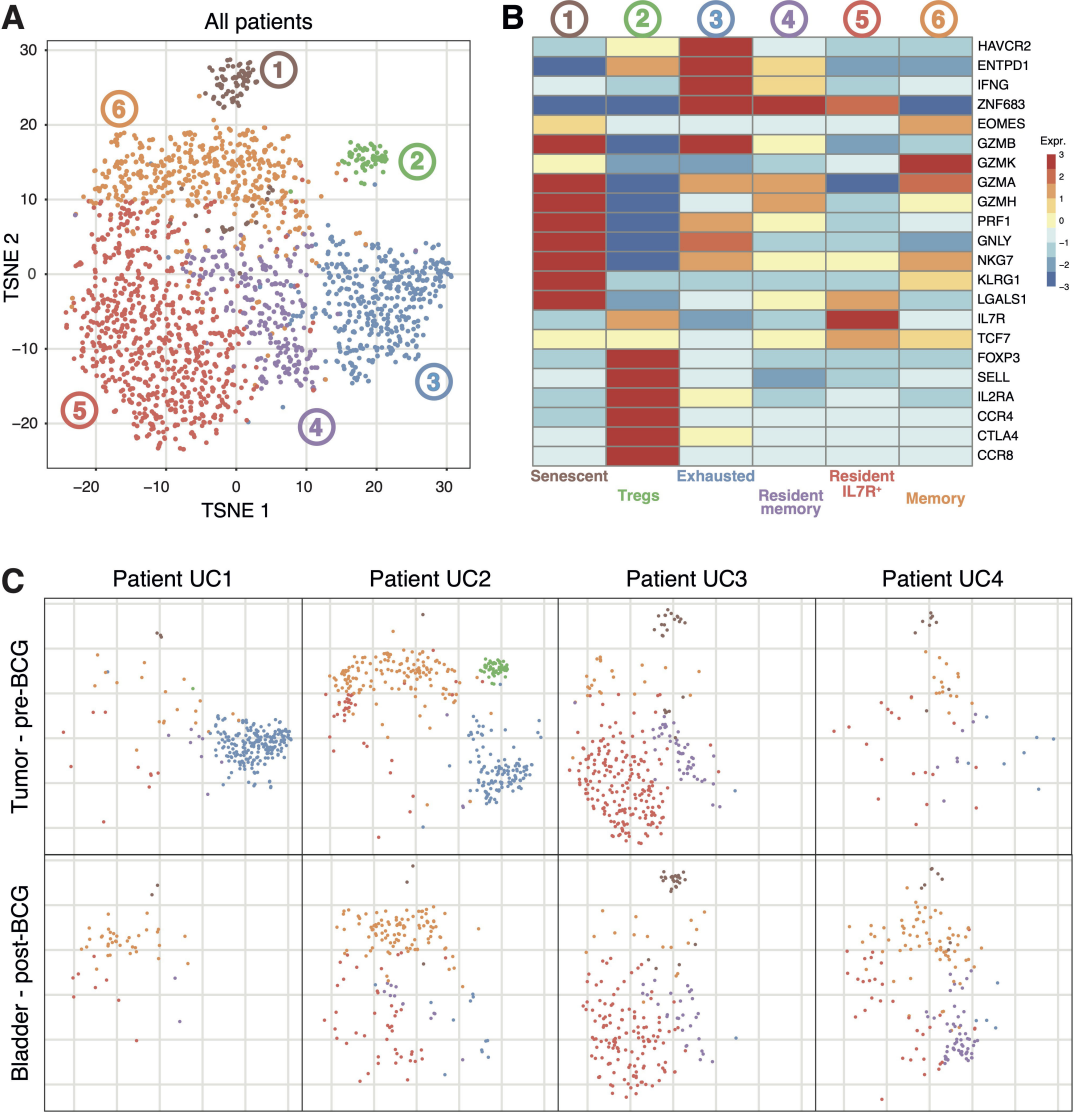
Transcriptomic profiles of CD8^+^ T cells extracted from the bladder of patients UC1 to UC4. **(A)** T-distributed stochastic neighbor embedding (t-SNE) projection of Smart-Seq2-based CD8^+^ single-cell data from 2040 CD8^+^ T cells extracted from the tumors and adjacent bladder tissues collected during TURBT, or from the same bladder regions 6 weeks after the last BCG instillation. **(B)** Heatmap of CD8^+^ T-cell clusters from **(A)** showing representative genes for each cluster. **(C)** Representations of **(A)** restricted to the indicated cell populations.

*The very small size of this sample precluded the sorting of T cells.

t-SNE dimensionality reduction analysis of CD8^+^ T cells from pre-BCG tumors and corresponding post-BCG bladder biopsies followed by graph-based clustering revealed 6 clusters (**Figure 2A**). First, cells qualified as senescent that express *KLRG1* but not *IL7R* (32) and strongly express cytotoxicity-associated genes such as *GZMB*, *GZMA* and *PRF1*. Second, a population of cells strongly expressing marker genes associated with T regulatory cells (Tregs) such as *FOXP3*, *IL2RA*, *CCR8* and *CTLA4*. Third, a population of exhausted T cells expressing *HAVCR2*, *ENTPD1*, *IFNG* and *ZNF683*, which encodes Hobit, a canonical transcription factor associated with tissue-residency. Next, two populations of resident cells expressing *ZNF683*, *CD69* and *ITGAE* (CD103), distinguished by the expression of *IL7R*. Finally, a population of memory cells that express *EOMES*, encoding the transcription factor eomesodermin, associated with T-cell memory differentiation (**Figure 2B**) (32).

Comparing these CD8^+^ populations in the tumors of the four patients revealed major differences. Exhausted cells were present only in the tumors of patients UC1 and UC2. The CD8^+^ Treg population was present only in the tumor of patient UC2. In the tumor of patient UC3, most of the cells were of the resident IL7R^+^ phenotype (**Figure 2C**). These results clearly show that each tumor contained functionally distinct populations of CD8^+^ T cells. Moreover, several of these populations were present in some tumors but absent from others. The causes of these important differences between tumors are unknown. Next, we compared these populations of pre-BCG CD8^+^ TILs with those present in the corresponding tumor-free bladder regions post-BCG (**Figure 2C**). The populations of exhausted and Treg CD8^+^ cells present in tumors of patients UC1 and UC2 were not detected anymore after BCG. In contrast, no clear difference between pre- and post-BCG samples was observed for the other CD8^+^ populations (**Figure 2C**). The results suggest that no new population of CD8^+^ T cells appeared in the bladder of these BCG-treated patients.

### Exploring the tumor-specificity of pre-BCG CD8^+^ TILs

Anti-neoepitope CD8^+^ T cells infiltrating metastatic melanomas, breast, colon and rectal tumors have been shown to be present mostly among exhausted T cell populations, presumably as a consequence of their chronic stimulation by tumor antigens (33, 34). As exhausted CD8^+^ T cells were clearly present in the tumor of patient UC1, we aimed to identify the antigens recognized by some of these cells (**Supplementary Figure 3**).

**Figure 3:**
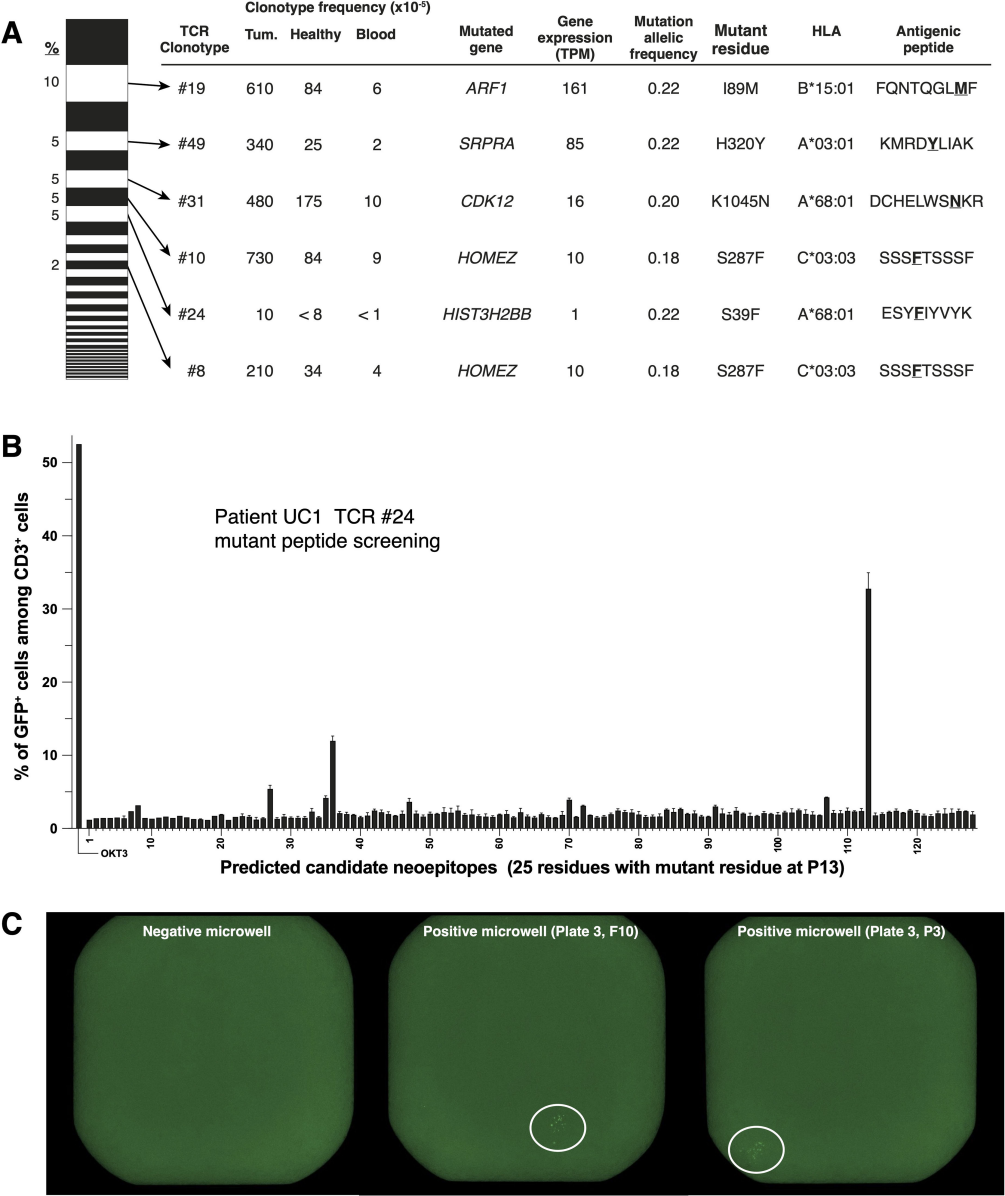
Identification of mutant peptides recognized by TCRs of CD8^+^ TILs from patient UC1. **(A)** On the left, TCR repertoire (48 TCR for 222 single T cells) of the ‘exhausted’ cluster shown on (**Figure 2**), ordered according to TCR frequencies. Six TCRs (#19, #49, #31, #10, #24 and #8), which were increased in tumor tissues compared to non-tumor tissues and blood, were screened for recognition of tumor antigens. **(B)** Example of peptide screening. Reporter Jurkat-2D3 expressing TCR #24 (15,000 cells/well) were incubated overnight with 30,000 autologous EBV-B cells pulsed with one of the indicated peptides. NFAT-GFP reporter gene activation was assessed with flow cytometry. **(C)** Example of cDNA library screening.

The TCR repertoire of the 222 exhausted CD8^+^ cells extracted from the tumor of patient UC1 consisted of 48 clonotypes (**Figure 3A**). Six of them, all enriched in tumor tissue as compared to non-tumoral adjacent pre-BCG bladder tissue and blood (**Figure 3A**), were screened for antigen recognition. Reconstructed TCR sequences were transduced into Jurkat 2D3 cells, which have lost endogenous TCR expression and express CD8ß and a NFAT reporter gene (35). TCR-transduced Jurkat 2D3 were screened for recognition of UC1 EBV-B cells incubated with a set of 125 synthetic mutant peptides predicted to bind to UC1 HLA class I molecules. Four TCRs recognized a mutant peptide (**Figure 3A**). TCR #10 and #8 recognized the same peptide. The results of one screening are shown on **Figure 3B**. TCRs #49 and #31 recognized none of the mutant peptides present in the tested set and were screened on a cDNA library prepared with mRNA extracted from UC1 tumoral tissue. One screening result is shown in **Figure 3C**. The two TCRs proved to recognize also a mutant peptide (**Figure 3A**). One of them, DCHELWSNKR presented by HLA-A*68:01 to TCR #31, was encoded by gene *CDK12*. *CDK12* mutations are frequently found in human tumors, including bladder cancer (36).

The CD8^+^ TILs of patient UC2 were functionally more diverse than those of patient UC1. We selected 6 TCRs from clusters ‘exhausted’, ‘Tregs’ and ‘memory’, none of which scored positive against a set of 50 mutant peptides containing epitopes predicted to bind to autologous HLA class I molecules. One clonotype, TCR #52 from the ‘memory’ cluster, recognized autologous EBV-B cells. Previous reports have identified such bystander T cells in human tumors (37). These negative results were surprising for the TCRs of the 5 ‘exhausted’ T cells and left open the possibility that they could recognize other tumor-specific antigen(s) than mutant peptides resulting from a single nonsynonymous nucleotide polymorphism. All 5 remaining TCRs were therefore screened against transfectants of a tumoral cDNA library (**Supplementary Table 2**). These TCRs did not appear to recognize tumor-specific antigens. For patient UC3, two clonotypes (#65 and #68) were expanded in the tumor of the patient. The TCRs of these clonotypes showed no reactivity against a set of 106 mutant peptides. No TCRs were selected for patient UC4, as no clonotype was enriched in the tumor of the patient compared to blood and adjacent normal tissue.

Concerning global TCR repertoire, we did not find clear patterns differentiating pre-and post-BCG PBMC samples, in terms of clonality index, V-gene usage, CDR3 length (data not shown). In addition, comparing pre-and post-BCG repertoires for each patient did not show global shifts in TCR frequencies (data not shown).

HLA class I expression is necessary for presentation of epitopes to CD8^+^ T cells. We performed beta-2-microglobulin (B2M) staining by immunohistochemistry, as a proxy for HLA class I expression, on the tumors of patients UC1, UC2 and UC3. Unfortunately, there was not enough tissue available to perform B2M staining for UC4 patient. We found that tumoral B2M expression was high in patient UC1, intermediate in patient UC2 and absent in patient UC3 (**Supplementary Figure 4**). These results led us to evaluate the status of the *HLA* and *B2M* loci, as genetic modifications of these genes are a frequent cause of altered HLA class I expression. No genetic anomaly was found for patient UC1 and UC3. A clear loss of heterozygosity at the HLA locus was detected in the tumor of patient UC2, providing an explanation for the reduced intensity of the B2M staining. We did not find a genetic alteration that could explain the undetectable HLA class I expression in the tumor cells of patient UC3.

In conclusion, from these analyses of CD8^+^ T cells present in NMIBC of 4 patients at the TURBT, we could demonstrate the presence of a spontaneous tumor-specific T cell response only in the tumor of patient UC1. All the tumor-specific CD8^+^ T cells that we analyzed (n=6) proved to recognize mutant peptides, which is in line with a higher tumor mutational burden in this tumor than in those of patients UC2, UC3 and UC4 (**Table 3**), and an intact HLA class I expression in the tumor. It is remarkable that by analyzing only 6 out of the 48 TCRs expressed by 222 CD8^+^ cells with an ‘exhausted’ phenotype we identified 5 different tumor-specific antigens, suggesting that many more tumor-specific antigens were immunogenic to CD8^+^ T cells in that tumor. These positive results are in sharp contrast with what we observed in the other two tumors, for which only negative results were obtained when screening sets of candidate mutant peptides as well as tumoral cDNA libraries.

### Impact of BCG on tumor-specific CD8^+^ T-cell responses

**Figure 4:**
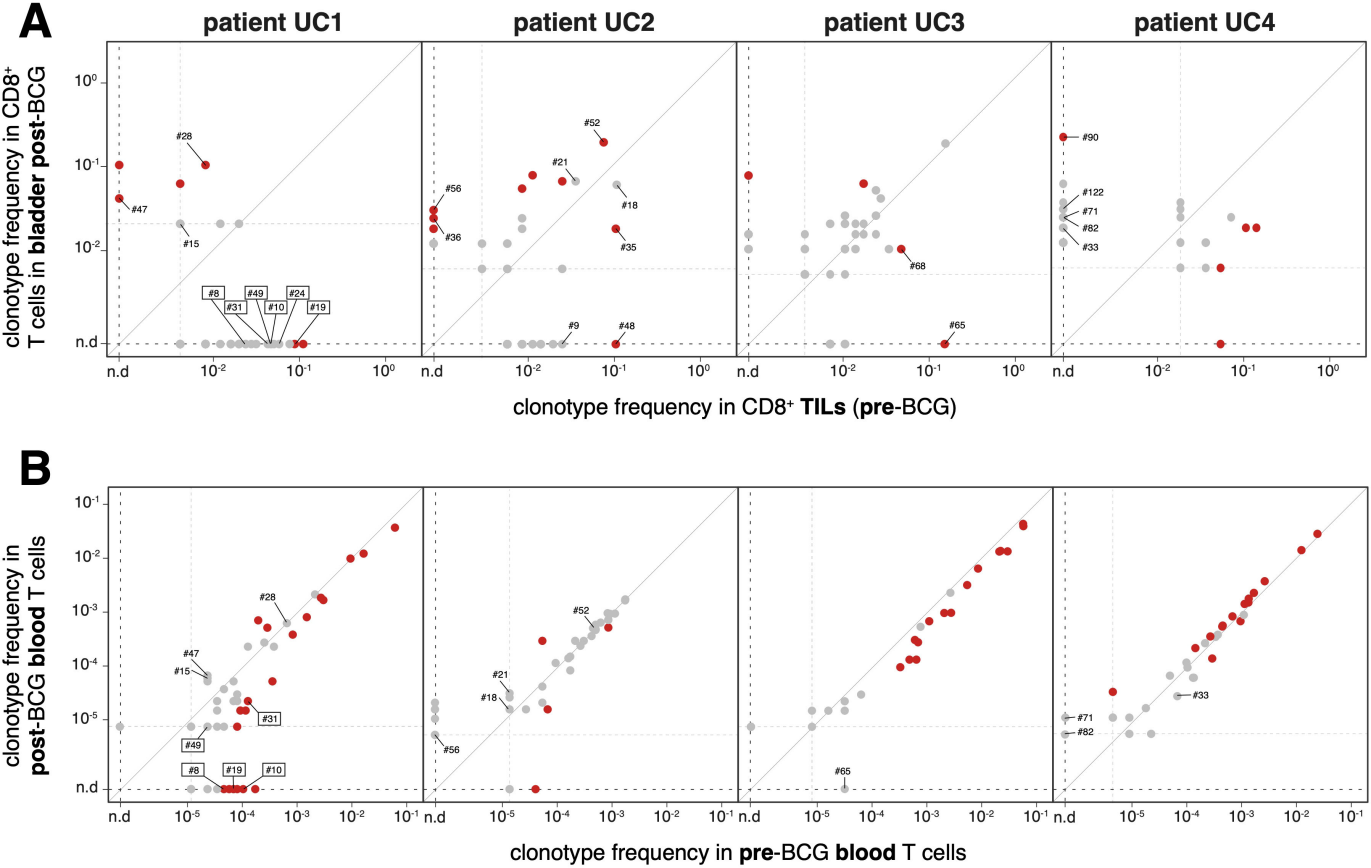
Frequencies of CD8^+^ T cell clonotypes in bladder and blood, pre- and post-BCG **(A)** Frequencies of TCR clonotypes were established on the basis of the *TRA* and *TRB* sequences identified in the single cell RNA-Seq data. Only clonotypes detected in at least two cells are shown. Those with statistically significant differences between pre-BCG and post-BCG frequencies are indicated in red (Fisher’s exact test, P < 0.05). Clonotypes identified with a number were screened for recognition of mutant peptides and of tumor cDNA library. Lowest frequency thresholds are indicated by dotted lines. For patient UC1 the TCR clonotypes with a demonstrated tumor-specificity are boxed. **(B)** Frequencies of the same TCR clonotypes in blood before and after therapy, established on the basis of *TRB* repertoires on blood DNA (Adaptive Biotechnologies).

The clinical benefit of adjuvant BCG instillations in the bladder could result from a local inflammatory response, which could contribute to either increase the frequency of existing tumor-specific CD8^+^ cytolytic T cells or prime new ones. We first addressed the potential boosting effect of the BCG therapy in patient UC1, who had mounted a spontaneous tumor-specific response detected in TILs prior to BCG treatment. In the bladder tissue collected 6 weeks after the last BCG instillation, the frequencies of the six tumor-specific TCRs had decreased at least 10-fold as compared to the tumor at the time of TURBT, even though the difference was statistically different only for clonotype #19 (**Figure 4A**). Considering that after the treatment there was no tumoral tissue left detected in the bladder biopsies, the absence of tumor-specific CD8^+^ TILs was expected. In addition, these tumor-specific T cells did not appear to persist in the bladder as tissue-resident memory T cells, which could participate in a long-term immunosurveillance of the primary tumor site. We also looked for an increase in the frequencies of tumor-specific CD8^+^ T cells in the blood of patient UC1, concomitant with BCG instillations. As shown in **Figure 4B**, 3 of the 6 clonotypes (#8, #10 and #19) were detected in pre-BCG blood but not anymore after BCG. Two other clonotypes had decreased blood frequencies after BCG. One tumor-specific clonotype (#24) was never detected in blood. Thus, for the tumor-specific CD8^+^ TILs of patient UC1, we have no element in favor of a local or systemic frequency increase concomitant with BCG instillations. Next, we examined whether in patient UC1 new CD8^+^ T cell clonotypes appeared after BCG. In the post-BCG bladder, the frequencies of 4 clonotypes increased significantly compared to the pre-BCG tumor. Two of these clonotypes (#28 and #47) were negative in our screenings for tumor specificity. We did not explore the specificity of the two other TCRs. For patients UC2, UC3 and UC4, some clonotypes had frequencies that increased significantly in the post-BCG bladder as compared to pre-BCG tumor. Three of them, #36 and #56 for patient UC2 and #90 for patient UC4, were tested for tumor specificity with negative results (**Supplementary Table 2**).

In conclusion, similarly to the cohort 1, our results demonstrate that tumor-specific CD8^+^ TILs in NMIBC can be spontaneously present with multiple specificities at the time of TURBT. However, this is not the case in all patients, as we detected no tumor-specific CD8^+^ T cells in three out of four examined tumors. In addition, in the patient with tumor-specific T cells, the frequencies of these T cells did not increase in blood or the bladder after the BCG instillations.

## Discussion

In this study, we first investigated the prevalence of tumor-specific CD8^+^ T cells in TILs and PBMCs from patients with non-metastatic bladder cancer. Along with melanoma and lung cancer, bladder cancer ranks amongst the most mutated cancers (38), resulting in a high number of neoepitopes and a correlation with the clinical response to ICT observed in metastatic bladder cancer (39). As in melanoma and lung cancer, patients with earlier-stage bladder cancer may also benefit from immunotherapy. However, the prevalence of spontaneous CD8^+^ T-cell responses in patients with non-metastatic bladder cancer had not been previously evaluated. In our study, across two cohorts, we detected neoantigen-specific CD8^+^ T cells in TILs or PBMCs from 10 out of 28 (~36%) non-metastatic bladder cancer patients. This proportion is likely underestimated, as spontaneous tumor-specific T-cell responses may fluctuate over time and could have been missed. Supporting this hypothesis, HLA class I alterations (indicative of immune selection pressure by T-cell responses) were reported in up to 72% of NMIBC cases (40). T-cell responses may also target other types of tumor-specific antigens. Notably, in the four patients from cohort 2, we used a tumor cDNA library screening method designed to detect a broader range of antigens. In patient 1, we identified two additional neoepitopes that had not been detected in the mutant peptide screenings; however, we did not identify other tumor-specific antigens.

In the NABUCCO trial (6), 24 patients with locally advanced bladder cancer received neoadjuvant ipilimumab and nivolumab. At cystectomy, 11 out of 24 (46%) patients achieved a pathological complete response. The proportion of patients with a preexisting tumor-specific T-cell response was not assessed in that study. Although anti-CTLA-4 antibodies can prime new tumor-specific T cells, it is plausible that most responders had a preexisting CD8^+^ T-cell response. It is likely that the spontaneous tumor-specific CD8^+^ T-cell responses we detected influence bladder cancer progression, even in the absence of immunotherapy. Supporting this hypothesis, our study provides the first evidence that MIBC patients with a detectable tumor-specific CD8^+^ T-cell response exhibited a lower rate of disease progression.

Tumor-specific CD8^+^ T cells may also play a crucial role in the mechanism of action of BCG, either by being boosted or by facilitating the induction of new responses during treatment. In this work, we conducted an in-depth analysis of a small number of patients but found no direct evidence supporting this hypothesis. This is particularly relevant in patient UC4, who had CIS and experienced tumor clearance during BCG treatment, yet no new tumor-specific CD8^+^ T-cell responses were detected. Additionally, patient UC3, whose tumor exhibited undetectable HLA class I expression, showed no recurrence after BCG treatment, suggesting an HLA-I-independent mechanism of action. An alternative explanation is that BCG-specific immune responses are central to treatment efficacy. BCG is known to be internalized by cancer cells through macropinocytosis (41). BCG-specific CD8^+^ T cells can emerge following BCG immunization (42). Of note, for BCG antigens to be presented on cancer cell surface HLA class I molecules, cross-presentation would be required. We investigated the presence of BCG-specific CD8^+^ T cells by screening a BCG cDNA library against HLA class I alleles and HLA-E (which presents mycobacterial peptides to T cells (43)), using selected TCRs from clonotypes emerging during treatment. However, we did not detect such responses (data not shown). It is worth noting that we did not assess the presence of unconventional T cells, such as mucosal-associated invariant T (MAIT) cells, natural killer T (NKT) cells, or γδ T cells, all of which are known to participate in immune responses against *Mycobacteria* (44, 45).

CD4^+^ T cells were not analyzed in this study, despite their well-established importance in BCG treatment. This population undergoes the most significant expansion during BCG therapy (46). In mouse models of bladder cancer treated with BCG, tumor-specific T cells are induced, and cured mice are protected against tumor rechallenge. This effect appears to be mediated by CD4^+^ T cells, primarily through IFN-γ production (15). IFN-γ interacts directly with tumor cells via the IFN-γ receptor, leading to CIITA expression, which has been shown to be required for treatment efficacy in an MHC-II-independent manner (47). However, this mechanism may be specific to the mouse model, which does not fully replicate human disease. In particular, the widely used MB49 orthotopic bladder cancer is highly aggressive, necessitating BCG instillations just two days post-tumor implantation. Despite this, over 50% of BCG-treated mice succumb to rapid disease dissemination, potentially facilitating the induction of tumor-specific immunity. BCG instillations induce BCG-specific CD4^+^ T cells in both mice (14) and humans (48). In mice, prior parenteral exposure to BCG markedly improves treatment efficacy (14). However, as previously noted, this may be relevant only in the context of the aggressive MB49 model. Human bladder tumors typically develop over months, meaning that a gradual immune response against BCG may not be a limiting factor. A critical consideration in this regard is the recommendation that patients with CIS and residual disease after an initial BCG induction course should receive a second course before being classified as BCG-unresponsive (49). Mechanistically, the recognition of BCG antigens by CD4^+^ T cells via HLA class II molecules expressed on cancer cells could be a key factor underlying the antitumor effect of BCG. Supporting this hypothesis, the requirement for direct tumor-BCG interaction (50) and the improved recurrence-free survival observed with prolonged maintenance therapy over multiple years (51) suggest a sustained immune engagement. While murine bladder carcinoma cells can present BCG antigens to BCG-specific CD4^+^ T cells *in vitro* (52), this remains to be demonstrated in human bladder cancer cells.

Most research on BCG and bladder cancer has been done in mouse models, and human data remain scarce. Despite being based on a small cohort of bladder cancer patients, our study highlights the need for further investigation into the role of anti-BCG adaptive immunity in human bladder cancer. A crucial next step would be to compare the immune responses of patients with BCG-responsive versus BCG-unresponsive tumors to identify the immune cells mediating BCG’s antitumor effects.

## Data Availability

The original contributions presented in the study are publicly available. These data can be found here: https://www.ega-archive.org/studies/EGAS50000001248, https://ega-archive.org/studies/EGAS50000001382, https://ega-archive.org/studies/EGAS50000001384, https://ega-archive.org/studies/EGAS50000001383.
