# Supplementary material for: Detection of spontaneous anti-neoepitope T-cell responses in non-metastatic bladder cancer patients
Source: Front Immunol. 2025 Nov 12;16:1627914. doi: 10.3389/fimmu.2025.1627914 (PMC12648094; doi:10.3389/fimmu.2025.1627914)
Supplement: Supplementary file 1 [file Table1.docx]

**Supplementary Table 1: Description of discovered HLA class-I neo-epitopes from the cohort 1**

| Patient | Gene | Full name | Mutation | HLA restriction* | Mutated sequence | WT sequence |
| --- | --- | --- | --- | --- | --- | --- |
| URO291 | TIPRL | TOR signaling pathway regulator | P229L | A*02:01 | SLMHVP**L**SL | SLMHVP**P**SL |
| URO291 | IQGAP3 | IQ Motif Containing GTPase Activating Protein 3 | Q976E | A*02:01 | YLAKLIF**E**M | YLAKLIF**Q**M |
| URO475 | NA | NA | NA | NA | PP2 | PP2 |
| URO483 | KNTC1 | Kinetochore Associated 1 | D607H | A*03:01 | KLCPWFKN**H** | KLCPWFKN**D** |
| URO516 | RXRA | Retinoid X Receptor Alpha | S427F | B*07:02 | LPALR**F**IGL | LPALR**S**IGL |
| URO671 | RPLP0 | Ribosomal Protein Lateral  Stalk Subunit P0 | R214C | B*18:01 | TEETLHS**C**F | TEETLHS**R**F |
| URO671 | RPLP0 | Ribosomal Protein Lateral  Stalk Subunit P0 | R214C | B*18:01 | EETLHS**C**F | EETLHS**R**F |
| URO774 | XRN1 | 5'-3' Exoribonuclease 1 | S539L | B*44:02 | NED**L**PIIEYY | NED**S**PIIEYY |
| URO774 | XRN1 | 5'-3' Exoribonuclease 1 | S539L | B*44:02 | NED**L**PIIEY | NED**S**PIIEY |
| URO786 | SMG9 | SMG9 Nonsense Mediated  MRNA Decay Factor | T361I | A*01:01 | GSDEG**I**EYY | GSDEG**T**EYY |
| URO845 | ITGA9 | Integrin Subunit Alpha 9 | E768K | C*05:01 | **K**VDTSITGI | **E**VDTSITGI |
| URO845 | NA | NA | NA | NA | PP7 | PP7 |
| URO878 | CLUH | Clustered Mitochondria Homolog | S342W | B*44:02 | AEDAYT**W**RL | AEDAYT**S**RL |
| URO878 | CLUH | Clustered Mitochondria Homolog | S342W | B*44:02 | AEDAYT**W**RLGY | AEDAYT**S**RLGY |
| URO878 | LGALS9 | Galectin 9 | F164L | B*35:05 | VP**L**SQPVCF | VP**F**SQPVCF |
| *Based on PRIME algorithm; PP = Peptide pool; NA = Not Applicable. | | | | | | |
