# Supplementary material for: Detection of spontaneous anti-neoepitope T-cell responses in non-metastatic bladder cancer patients
Source: Front Immunol. 2025 Nov 12;16:1627914. doi: 10.3389/fimmu.2025.1627914 (PMC12648094; doi:10.3389/fimmu.2025.1627914)
Supplement: Supplementary file 2 [file Table2.docx]

**Supplementary Table 2:**

**Results of peptide and cDNA library screenings for the patients of cohort 2.**

|  | |  | |  |  | **Screened mutant peptides** | | **Screened cDNAs** | | | | | | | |  |  |
| --- | --- | --- | --- | --- | --- | --- | --- | --- | --- | --- | --- | --- | --- | --- | --- | --- | --- |
|  | |  | |  |  |  |  |  |  |  |  |  |  |  |  |  |  |
| **Patient** | | **TCR** | | **Phenotype** | **Category** | **Nb**  **peptides** | **+** | **HLA** | **Nb+ wells /tested** | **HLA** | **Nb+ wells /tested** | **HLA** | **Nb+ wells /tested** | **HLA** | **Nb+ wells /tested** | **Note** |  |
|  | |  | |  |  |  |  |  |  |  |  |  |  |  |  |  |  |
|  | |  | |  |  |  |  |  |  |  |  |  |  |  |  |  |  |
| **UC1** | | #8 | | Exhausted | pre-BCG | 128 | **+** | C3 | 2/7680 |  |  |  |  |  |  | a |  |
|  | | #10 | | Exhausted |  | 128 | **+** |  |  |  |  |  |  |  |  |  |  |
|  | | #19 | | Exhausted |  | 128 | **+** | B15 | 341/7680 |  |  |  |  |  |  | a |  |
|  | | #24 | | Exhausted |  | 128 | **+** | A68 | 0/7680 |  |  |  |  |  |  | b |  |
|  | | #31 | | Exhausted |  | 128 | - | A68 | 18/7680 |  |  |  |  |  |  |  |  |
|  | | #49 | | Exhausted |  | 128 | - | A3+B15 | 8/3840 | A68+B35 | 0/3840 |  |  |  |  |  |  |
|  | | #15 | | Res mem |  | 128 | - | A3+B15 | 0/3840 | A68+B35 | 0/3840 | C3 | 0/3840 | C4 | 0/3840 |  |  |
|  | | #28 | | Res mem | post-BCG | 128 | - | A3+B15 | 8/3840 | A68+B35 | 0/3840 | C3 | 0/3840 | C4 | 0/3840 | c |  |
|  | | #47 | | Memory |  | 128 | - | A3+B15 | 0/3840 | A68+B35 | 0/3840 | C3 | 0/3840 | C4 | 0/3840 |  |  |
|  | |  | |  |  |  |  |  |  |  |  |  |  |  |  |  |  |
|  | |  | |  |  |  |  |  |  |  |  |  |  |  |  |  |  |
| **UC2** | | #9 | | CD8^+^ Treg | pre-BCG | 50 | - | A3+C15 | 13/7680 |  |  |  |  |  |  | c |  |
|  | | #18 | | Exhausted |  | 50 | - | A3+C15 | 0/7680 | A29 | 0/7680 | B44 | 10/7680 | B51+C16 | 0/3840 | d |  |
|  | | #21 | | Exhausted |  | 50 | - | A3+C15 | 0/7680 | A29 | 0/7680 | B44 | 0/3840 | B51+C16 | 0/3840 |  |  |
|  | | #35 | | Exhausted |  | 50 | - | A3 | 3/7680 |  |  | B44 | 0/3840 | B51+C16 | 0/3840 | c |  |
|  | | #48 | | Exhausted |  | 50 | - | A3+C15 | 0/7680 | A29 | 0/7680 | B44 | 0/3840 | B51+C16 | 0/3840 |  |  |
|  | | #56 | | Exhausted | post-BCG | 50 | - | A3+C15 | 0/7680 | A29 | 0/7680 | B44 | 0/3840 | B51+C16 | 0/3840 |  |  |
|  | | #36 | | Res IL7R+ |  | 50 | - | A3+C15 | 0/7680 | A29 | 0/7680 | B44 | 0/3840 | B51+C16 | 0/3840 |  |  |
|  | | #52 | | Memory | pre-BCG | 50 | - | A3+C15 | 0/7680 | A29 | 0/7680 | B44 | 0/3840 | B51+C16 | 0/3840 | e |  |
|  | |  | |  |  |  |  |  |  |  |  |  |  |  |  |  |  |
|  | |  | |  |  |  |  |  |  |  |  |  |  |  |  |  |  |
| **UC3** | | #65 | | Res mem | pre-BCG | 106 | - |  |  |  |  |  |  |  |  |  |  |
|  | | #68 | | Res IL7R+ |  | 106 | - |  |  |  |  |  |  |  |  |  |  |
|  | |  | |  |  |  |  |  |  |  |  |  |  |  |  |  |  |
|  | |  | |  |  |  |  |  |  |  |  |  |  |  |  |  |  |
| **UC4** | | #82 | | Res mem | post-BCG | 160 | - | A3+B18 | 0/3840 | A24+B55 | 0/3840 | C3 | 0/3840 | C12 | 0/3840 |  |  |
|  | | #90 | | Res mem |  | 160 | - | A3+B18 | 0/3840 | A24+B55 | 0/3840 | C3 | 0/3840 | C12 | 0/3840 |  |  |
|  | | #33 | | Res IL7R+ |  | 160 | - |  |  |  |  |  |  |  |  |  |  |
|  | | #71 | | Res IL7R+ |  | 160 | - | A3+B18 | 0/3840 | A24+B55 | 0/3840 | C3 | 0/3840 | C12 | 0/3840 |  |  |
|  | | #122 | | Memory |  | 160 | - | A3+B18 | 0/3840 | A24+B55 | 0/3840 | C3 | 0/3840 | C12 | 0/3840 |  |  |
|  | |  | |  |  |  |  |  |  |  |  |  |  |  |  |  |  |
| a |  | | These results confirmed the validity of the screening method, as we were able to detect the cDNA fragments encoding the mutant peptides that  were shown to stimulate these TCRs in the peptide library screenings. | | | | | | | | | | | | | | |
| b |  | | This screening was negative even though this TCR recognizes a mutant tumor antigen. We found that the gene encoding this antigen was  expressed at a very low level (1 TPM), causing the cDNA fragment to be at a very low frequency in the cDNA library. | | | | | | | | | | | | | | |
| c |  | | cDNAs that contain no mutation and are ubiquitously expressed, with putative antigenic peptides not completely characterized. | | | | | | | | | | | | | | |
| d |  | | Cross-reactivity on a chimeric peptide encoded partly by an adapter sequence. | | | | | | | | | | | | | | |
| e |  | | This TCR is activated by autologous EBV-B cells and probably recognizes an EBV antigen. | | | | | | | | | | | | | | |
|  |  | |  | | | | | | | | | | | | | | |
