# Supplementary material for: Detection of spontaneous anti-neoepitope T-cell responses in non-metastatic bladder cancer patients
Source: Front Immunol. 2025 Nov 12;16:1627914. doi: 10.3389/fimmu.2025.1627914 (PMC12648094; doi:10.3389/fimmu.2025.1627914)

Supplementary Figure 1: Functional analysis of neoantigen-specific CD8^{+} T cells. (A) Representative example of CD107a, IFN-γ, TNF-α and IL-2 intracellular labelling in live CD3^{+}CD8^{+} T cells upon neopeptide stimulation from patient URO516. (B) CD107a, IFN-γ, TNF-α and IL-2 expression profiles from TIL from 6 patients upon neoepitope stimulation.

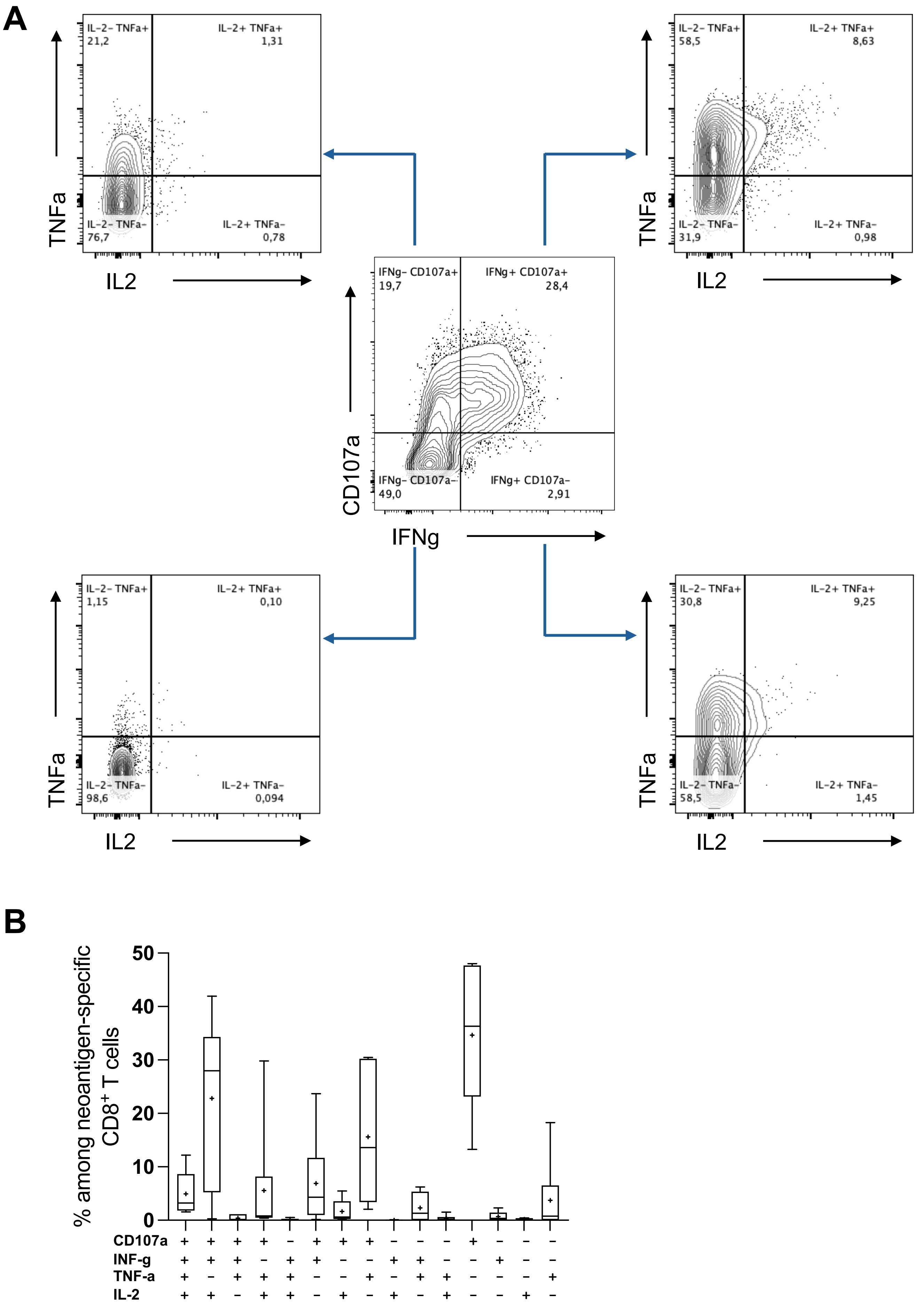

Supplementary Figure 2: Recurrence and progression free-survival based on the presence of detectable reactivity against neoantigen in non-muscle (NMIBC) and muscle invasive bladder cancer patients (MIBC)

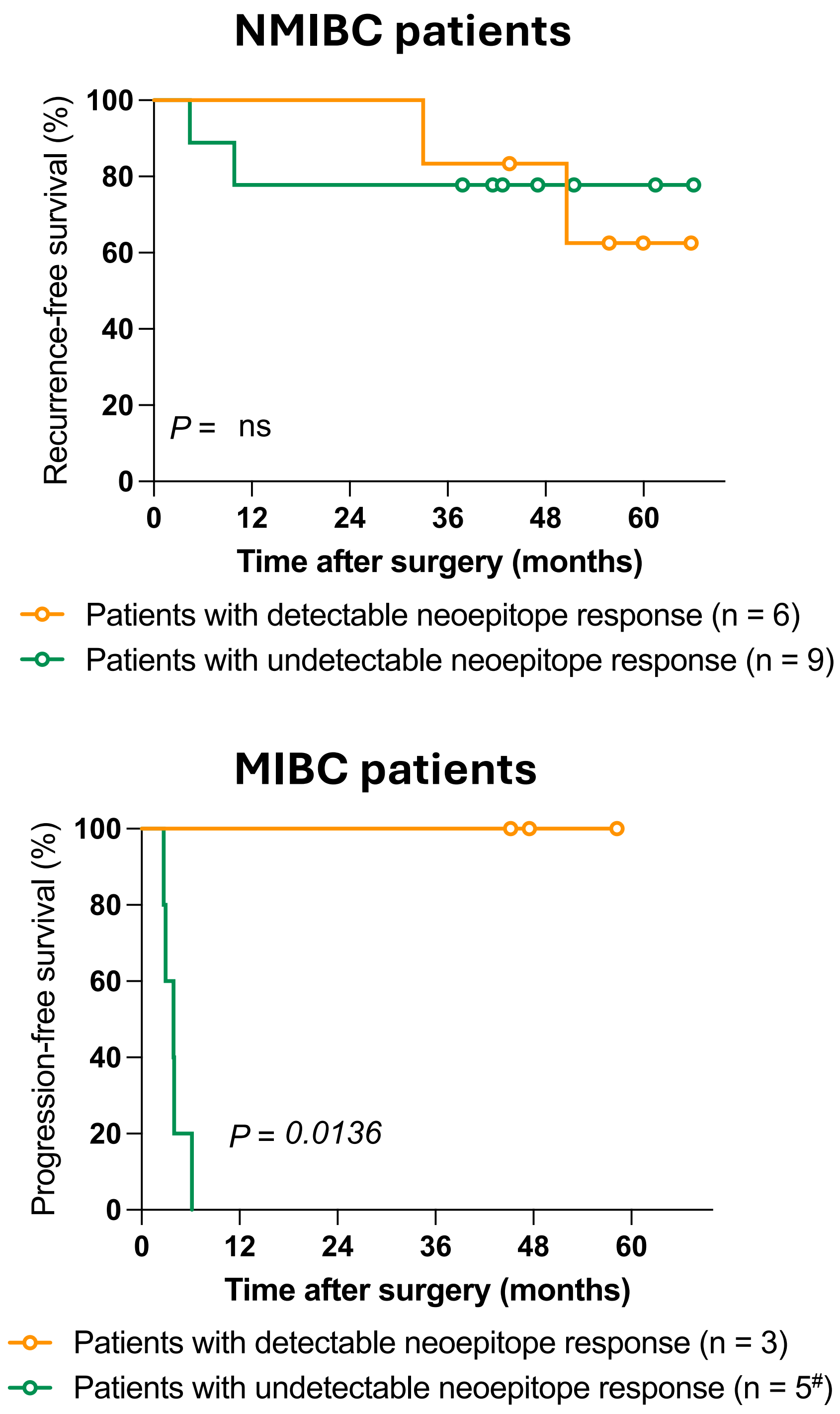

No clinical follow-up for one patient

Supplementary Figure 3: Overview of the cDNA library construction and screening strategy.

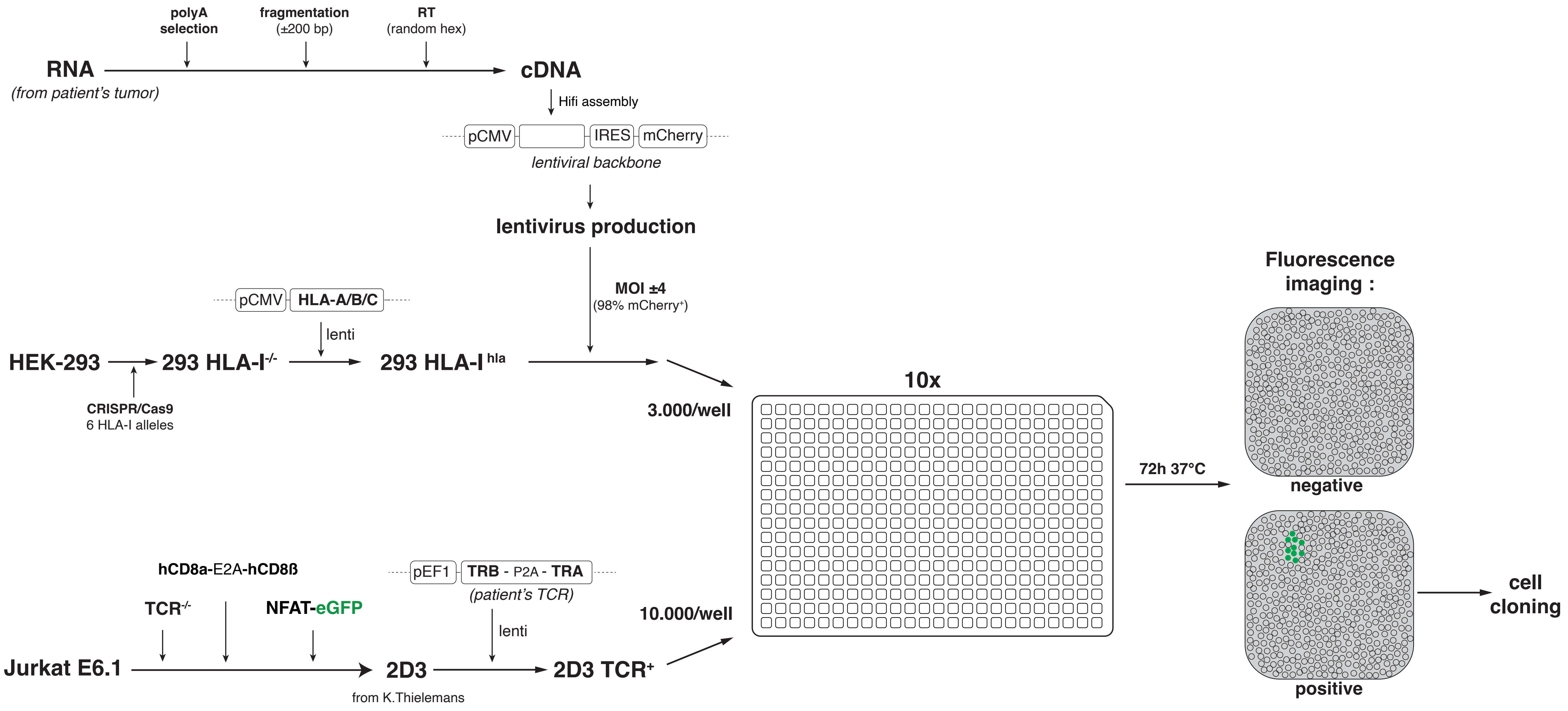

Supplementary Figure 4: Immunohistochemical detection of Beta-2-Microglobulin in the tumors of patients UC1 to UC3.

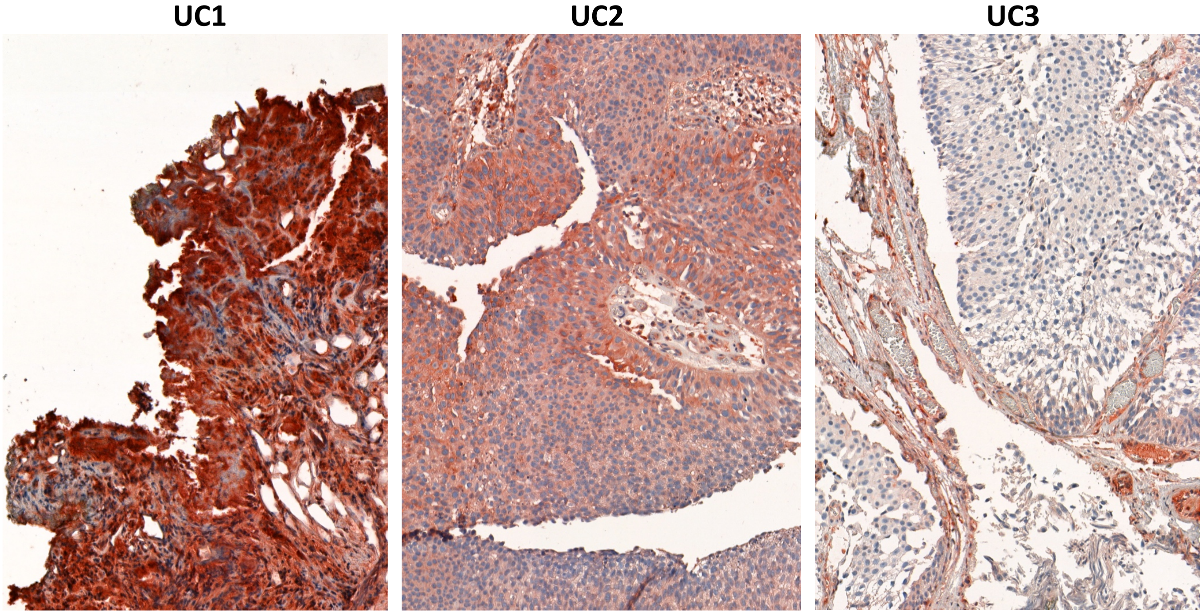

Supplement: Supplementary file 3 [file DataSheet1.pdf]
