# Supplementary material for: Detection of spontaneous anti-neoepitope T-cell responses in non-metastatic bladder cancer patients
Source: Front Immunol. 2025 Nov 12;16:1627914. doi: 10.3389/fimmu.2025.1627914 (PMC12648094; doi:10.3389/fimmu.2025.1627914)
Supplement: Supplementary file 4 [file DataSheet2.docx]

**SUPPLEMENTARY METHODS FOR PATIENTS OF COHORT 2**

**Peptide recognition assays**

Peptides were synthesized by Genscript (NJ, USA) as lyophilized powder, resuspended in DMSO and stored at -20°C until further use. For peptide screenings, 30.000 EBV-B cells were pre-incubated with single peptides for 2 h in IMDM 1% FCS in U-bottom or flat-bottom 96-well plates. Next, two TCR-Jurkat cell lines were added (15.000/cell line) in IMDM 10% FCS. Final concentration of the peptide was 10 µM. Co-incubation was performed overnight at 37°C 8% CO_2_. As positive controls, Jurkat cells were stimulated with a coated anti-CD3 antibody (clone OKT3, Bio X Cell) or a bispecific anti-CD3/anti-CD19 antibody (Blinatumomab, Invivogen). The percentage of GFP^+^ Jurkat cells was measured by flow cytometry (LSRFortessa, BD Biosciences) or with an automated cell imaging system (ImageXpress Pico, Molecular Devices).

***TCRB* repertoires**

*TCRB* repertoires were established by Adaptive Biotechnologies (Seattle, USA) starting from DNA purified from PBMCs (Deep resolution) or tissue samples (Survey resolution).

**Lentivirus production**

On day 0, 7.5 10^5^ HEK-293T cells were plated in 6-well plates (Corning, MA, USA). On day 1, the cells were transfected with 0.9 µg of transfer plasmid, 0.6 µg of pcCMV-VSVg (Addgene, #8454) and 1 µg of psPAX2 (Addgene, #12260) using TransIT-LT1 (Mirus Bio, WI, USA). On day 4, supernatant was harvested, filtered on 0.45 µm, and concentrated 20x on a Vivaspin centrifugal concentrator (Sartorius, #VS0642).

**Generation of TCR-transduced Jurkat reporter cells**

For selected TCRs, sequences encoding the TCR-α and β chains were synthesized and cloned with a GSG-P2A linker into pcDNA3.1 (Synbio Technologies, NJ, USA). The complete TCR sequence was transferred in a lentiviral vector downstream of an EF-1 promoter. Recombinant viruses were used to transduce the Jurkat 2D3 cells, produced from the TCR α/ß-deficient Jurkat-76 cells (1) by transfection first of a construct encoding CD8α and CD8ß and then of a NFAT-eGFP reporter gene (2). Jurkat-2D3 (10^5^cells per well) were spin-infected for 30 min at 800 g in flat-bottom 96-well plates (Corning, MA, USA) in RPMI (Gibco, ThermoFisher Scientific) with 10% FCS (Fetal Calf Serum, Sigma #F7524 or Cytiva #SV30160.03), 8 µg/ml Polybrene (Merck, #TR-1003) and incubated for 24 h. TCR expression was verified by staining with an anti-TCR-APC (IP26) antibody (Biolegend) and populations of 100% TCR^+^ Jurkat-2D3 cells were sorted by flow cytometry.

**EBV-transformed B cell line generation**

For each patient, 2-9 10^6^ PBMCs were thawed and co-cultured in 1 ml of IMDM 10% FCS with 10^5^ 3T6 cells stably transfected with a *CD40L* construct (in-house production), with 1 ml of B95-8 culture supernatant containing EBV (in house production) and 1 µg/ml cyclosporin A (Novartis, Basel, Switzerland) for 4 weeks. Fresh medium with cyclosporin A was added as needed. Cells were expanded in cyclosporin-free medium and cryopreserved until further use.

**Generation of HLA-transduced HEK-293T cells**

HEK-293T cells were first knocked-out for endogenous HLA class I alleles. Sequences shared between all the class I HEK 293T alleles were identified. A target sequence in the alpha-2 domain was selected (5’-GATGTAATCCTTGCCGTCGT-3’). Complementary oligonucleotides corresponding to the target sequence were synthesized, annealed and cloned into a pX458 backbone encoding Cas9. 10^5^ HEK-293T cells were transfected with 0,25 µg vector using TransIT-LT1 in a 48-well plate. Next, HLA expression was evaluated by staining with the anti-HLA-A,B,C-APC antibody W6/32 (Biolegend). HLA-negative cells were cloned and after confirmation of the absence of HLA expression, a clone was selected and cryopreserved for further use. HLA alleles were synthesized as gBlocks (Integrated DNA Technologies, IA, USA) and cloned in a lentiviral vector downstream of a CMV promoter. After transduction, HLA expression was verified by antibody staining and cells were sorted by FACS to obtain 100% expression. Anti-HLA antibodies

were anti-HLA-A,B,C-Alexa Fluor 647 (W6/32(, anti-HLA-A2-FITC (BB7.2), anti-HLA-E-PE (3D12) (Biolegend), anti-HLA-A3-BV421 (GAP.A3), anti-HLA-C-PE (DT-9) (BD Biosciences), anti-HLA-A2,A28-PE (REA142), anti-HLA class I Bw4-APC (REA274),  anti-HLA class I Bw6-APC (REA143) (Miltenyi Biotec) and anti-HLA-A24 (AH254, in house production).

**Antibody panel for T cell isolation for scRNAseq**: anti-CD3-FITC (UCHT1), anti-CD8-PEdazzle594 (RPA-T8), anti-CD45-Alexa Fluor 700 (HI30), anti-CD25-BV785 (BC96), anti-CD69-Alexa Fluor 647, anti-GARP-PE (7B11), anti-PD1-BV421 (EH12.2H7), anti-CD103-BV711 (Ber-ACT8), anti-CD56-PE/Cy7 (3G8), anti-CD16-PerCP/Cy5.5, anti-CD19-BV650 (HIB19) (all from Biolegend) anti–CD4-BV480 (RPA-T4), anti-CD33-BV605 (both from BD Biosciences) and Fixable viability dye-eFluor 780 (ThermoFisher Scientific, #65-0865-14).

**cDNA library synthesis and screening (Supplementary Figure 2)**

RNA integrity was assessed by electrophoresis (Tapestation RNA, Agilent). Starting from 0.1-1 µg of RNA, a cDNA library was constructed using the NEBNext Ultra II Directional RNA Library Prep kit (NEB, #E7765), using the poly(A) mRNA Isolation Module (NEB, #E7490S). RNA was fragmented by incubation with Mg^2+^ at 94°C and reverse transcribed with random hexamers. After adaptor ligation, the fragments were amplified with oligonucleotides 5’-AATCGTATGGGACTG

GAGTTCAGACGTGT-3’ and 5’-TTATTCAGTTACACTCTTTCCCTACACGAC-3’ and cloned into a lentiviral vector (promCMV, IRES-mCherry) by HiFi assembly (NEB, #E2621) (Fig. S4). The vector library was cleaned-up with AMPure XP beads, eluted in 4 µl of water and electroporated in Endura electrocompetent cells (Lucigen, WI, USA). The bacteria were plated on LB agar on 20 Nunc Square BioAssay Dishes (ThermoFisher Scientific, #240835), incubated at 32°C for 16 h and pooled. The plasmidic library was purified by Maxiprep (ThermoFisher Scientific, #K210007), eluted in TE and quantified by spectrophotometry. Next, lentiviral particles were produced in batches of 24 wells of 6-well plates and cryopreserved as aliquots of 400 µl of 20x-concentrated supernatant. One aliquot was used to determine the relative titer of the virus stock: HEK-293 cells were transduced with different viral concentrations and transduction efficacy assessed the following day by measuring the proportion of mCherry^+^ cells by flow cytometry.

To screen the cDNA libraries, HLA-transduced HEK 293T cells were transduced with a cDNA library containing lentiviral particles, with a target mCherry expression of 98% (MOI ±4). At day 1, the cells were dissociated with TrypLE Express (Thermofisher Scientific) and resuspended in complete IMDM medium. In ten flat-bottom µClear 384-well plates (Greiner), 3.000 library-transduced HEK cells were co-cultured with two TCR-Jurkat cell lines (5.000/cell line) in IMDM 10% FCS for 72 h at 37°C 8% CO2, after which fluorescence imaging of each well was performed on an automated cell imaging system (ImageXpress Pico, Molecular Devices) (Supplementary Figure 2). The images were manually browsed and wells with a putative hit (defined as a cluster of >8 GFP^+^ cells) were identified. For each selected well, cells were dissociated and replated in a 384-well plate. After 5 days, 5.000 TCR-Jurkat cells/well were added, each cell line in half of the plate, to determine which of the two TCRs was stimulated and to validate the putative hits. Cells were co-cultured for a supplementary 72 h after which images were re-acquired. For wells with GFP^+^ cells, cells were dissociated, amplified and mCherry^+^ cells were cloned in 384-well plates. After 7 days, 5.000 TCR-Jurkat cells/well were added, cells were co-incubated for 72 h and images acquired. In wells with GFP^+^ cells, the cells were dissociated, amplified and cryopreserved. Sequencing libraries were prepared from the selected clones. For each clone, ±50.000 cells were lysed in H20 and boiled at 100°C for 10 min, then treated with proteinase K (10 µg/ml) at 37°C for 1 h followed by 10 min at 95°C for enzyme inactivation. Next, lentiviral-inserted transgenes were amplified by PCR with oligonucleotides 5’ GACTGGAGTTCAGACGTGTGCTCTTCCGAT-CT 3’ and 5’ ACACTCTTTCCCTACACG -ACGCTCTTCCGATCT 3’, bead-purified (AMPure XP, Beckman Coulter), quantified (Quantifluor, Promega), normalized, after which index were added (NEBNext Multiplex Oligos for Illumina (#E6440, NEB). Libraries were pooled, purified (PCRapace, INVITEK Molecular) then quantified and quality-checked by electrophoresis (TapeStation, Agilent). Libraries were sequenced on MiSeq (Illumina). Sequencing reads were aligned to the human reference genome hg38 using Rsubread. Gene expression quantification was obtained with featureCounts. The cDNA fragment encoding the recognized antigen was identified as the fragment shared between clones originating from different wells in which a positive signal was identified at the end of the first co-culture round.

**Immunohistochemistry**

Tissue sections (4-5 µm thick) cut from formalin-fixed paraffin embedded tumor samples were mounted on microscope slides. All the procedures were carried out at room temperature. The sections were deparaffinized and rehydrated, and antigen retrieval was performed in citrate buffer at pH6 in a microwave oven. Endogenous peroxidases were blocked with Peroxidase Blocking Reagent (Agilent) for 10 minutes, then unspecific antibody binding was blocked with Tris-buffered saline supplemented with 2% non-fat dehydrated milk, 5% bovine serum albumin, 1% human serum immunoglobulins and 0.15% Tween20 for 30 minutes. The sections were incubated for 1 hour with either an anti-B2M rabbit monoclonal antibody (clone D8P1H, Cell Signaling Technology) diluted 1:2000 or an anti-HLA class I heavy chain mouse monoclonal antibody (clone EMR8-5, Cell Signaling Technology) diluted 1:1000. The sections were washed and incubated with a species-matched secondary polyclonal goat antibody coupled to horseradish peroxidase (EnVision+ System-HRP Labelled Polymer Anti-Rabbit or anti-Mouse, Agilent) undiluted. The sections were stained with the 3-amino-9-ethylcarbazole (red staining) detection dye and counterstained with hematoxylin. For each tumor section analyzed, one adjacent section was processed identically but without the primary antibody step, as a negative control. High-resolution digital images from stained sections were acquired with a Pannoramic P250 Flash III slide scanner (3DHISTECH).

**Supplementary references.**

1. Heemskerk M.H. *et al.* Redirection of antileukemic reactivity of peripheral T lymphocytes using gene transfer of minor histocompatibility antigen HA-2-specific T-cell receptor complexes expressing a conserved alpha joining region. Blood **102**, 3530-3540 (2003).

2. Morimoto S. *et al.* Establishment of a novel platform cell line for efficient and precise evaluation of T cell receptor functional avidity. Oncotarget **75**:34132-34141 (2018).
